# Evaluation of Tetrahydrobiopterin Therapy with Large Neutral Amino Acid Supplementation in Phenylketonuria: Effects on Potential Peripheral Biomarkers, Melatonin and Dopamine, for Brain Monoamine Neurotransmitters

**DOI:** 10.1371/journal.pone.0160892

**Published:** 2016-08-11

**Authors:** Shoji Yano, Kathryn Moseley, Xiaowei Fu, Colleen Azen

**Affiliations:** 1 Genetics Division, Department of Pediatrics, LAC+USC Medical Center, Keck School of Medicine, University of Southern California, Los Angeles, CA, United States of America; 2 Department of Pathology, Children’s Hospital Los Angeles, Keck School of Medicine, University of Southern California, Los Angeles, CA, United States of America; 3 Clinical and Translational Science Institute, Keck School of Medicine, University of Southern California, Los Angeles, CA, United States of America; Pennsylvania State University College of Medicine, UNITED STATES

## Abstract

**Background:**

Phenylketonuria (PKU) is due to a defective hepatic enzyme, phenylalanine (Phe) hydroxylase. Transport of the precursor amino acids from blood into the brain for serotonin and dopamine synthesis is reported to be inhibited by high blood Phe concentrations. Deficiencies of serotonin and dopamine are involved in neurocognitive dysfunction in PKU.

**Objective:**

(1) To evaluate the effects of sapropterin (BH4) and concurrent use of large neutral amino acids (LNAA) on the peripheral biomarkers, melatonin and dopamine with the hypothesis they reflect brain serotonin and dopamine metabolism. (2) To evaluate synergistic effects with BH4 and LNAA. (3) To determine the effects of blood Phe concentrations on the peripheral biomarkers concentrations.

**Methods:**

Nine adults with PKU completed our study consisting of four 4-week phases: (1) LNAA supplementation, (2) Washout, (3) BH4 therapy, and (4) LNAA with BH4 therapy. An overnight protocol measured plasma amino acids, serum melatonin, and 6-sulfatoxymelatonin and dopamine in first void urine after each phase.

**Results:**

(1) Three out of nine subjects responded to BH4. A significant increase of serum melatonin levels was observed in BH4 responders with decreased blood Phe concentration. No significant change in melatonin, dopamine or Phe levels was observed with BH4 in the subjects as a whole. (2) Synergistic effects with BH4 and LNAA were observed in serum melatonin in BH4 responders. (3) The relationship between serum melatonin and Phe showed a significant negative slope (p = 0.0005) with a trend toward differing slopes among individual subjects (p = 0.066). There was also a negative association overall between blood Phe and urine 6-sulfatoxymelatonin and dopamine (P = 0.040 and 0.047).

**Conclusion:**

Blood Phe concentrations affected peripheral monoamine neurotransmitter biomarker concentrations differently in each individual with PKU. Melatonin levels increased with BH4 therapy only when blood Phe decreased. Monitoring peripheral neurotransmitter metabolites may assist in optimizing individualized treatment in PKU.

## Introduction

Since newborn screening has been introduced, early diagnosis and dietary management based on blood phenylalanine (Phe) concentrations successfully prevented individuals with Phenylketonuria (PKU; OMIM 261600) from developing significant intellectual disability. Recent studies, however, report that even individuals who are early diagnosed and well controlled have a high prevalence of neurocognitive and neuropsychological symptoms which include decreased executive functioning and internalizing disorders [1–3]. The dietary treatment of PKU is based on the restriction of Phe intake in order to maintain blood Phe concentrations within the recommended range. Blood Phe between 120 to 360 μM has been widely used as the recommended range for children younger than age 12 years and lower than 900 μM for individuals older than 12 years of age [4]. Recently, the American College of Medical Genetics has recommended blood Phe concentrations be maintained between 120 to 360 μM for all individuals with PKU in order to reduce the long term neurocognitive and neuropsychological symptoms [3]. Abnormal neurotransmitter metabolism, particularly serotonin and dopamine deficiency, are reported in PKU due to high blood Phe concentrations [5,6]. Most of those symptoms are considered to be related to the metabolism of monoamine neurotransmitters which include serotonin and dopamine [7, 8]. Tryptophan (Trp) and tyrosine (Tyr) are the precursor amino acids for serotonin and dopamine, respectively. They are transported into the central nervous system (CNS) by the large neutral amino acid (LNAA) transporter, LAT1 (SLC7A5) which transports the LNAA including branched chain (valine, leucine, and isoleucine), aromatic (Phe, Tyr, and Trp), and other amino acids (histidine, threonine, and methionine) [9]. Trp and Tyr compete with Phe at LAT1 in PKU. Although blood Trp and Tyr concentrations affect serotonin and dopamine synthesis in the brain, they are not usually monitored along with Phe to adjust dietary therapy as they are difficult to interpret due to significant fluctuations [10].

Some individuals with PKU with excellent control in blood Phe may still be deficient in serotonin and dopamine in the CNS, particularly with restriction of natural protein and/or insufficient use of medical food products containing LNAA including Trp and Tyr [6,11]. Conversely, other individuals with blood Phe above the recommended range may have normal serotonin and dopamine metabolism in the CNS; it is well known that some do very well despite blood Phe concentrations significantly above the recommended range.

Evaluation of monoamine neurotransmitter metabolism in the CNS could provide essential information to optimize dietary management for individuals with PKU. However, it is impractical to directly monitor the cerebrospinal fluid (CSF) serotonin and dopamine metabolites, i.e., 5-hydroxyindoleacetic acid (5HIAA) and homovanillic acid (HVA), respectively, due to the invasive nature of the procedure. Therefore, we conducted studies evaluating peripheral melatonin and dopamine in individuals with PKU based on the hypothesis that these potential biomarkers reflect serotonin and dopamine metabolism in the CNS. Our studies to date are consistent with findings by Nielsen and Lykkelund demonstrating increased concentrations of monoamine neurotransmitter metabolites, i.e., 5HIAA and HVA in the CSF in individuals with PKU after implementation of the Phe restricted diet or after supplementation of the precursor amino acids [6,12,13,14].

Since melatonin is synthesized from serotonin in the pinealocytes, we measured this metabolite as a biomarker to reflect serotonin synthesis in the brain in our previous studies [12,13]. These studies showed significantly low melatonin concentrations in blood and urine in adult individuals with PKU, due to two reasons secondary to high blood Phe concentration: (1) competitive inhibition of Trp transport at LAT1, and (2) inhibition at Trp hydroxylase (TPH, EC 1.14.16.4). Melatonin synthesis was significantly increased, although not normalized, by LNAA supplementation providing Trp at 30 mg/kg/d, without a significant reduction of blood Phe [12]. Urine dopamine was increased by Tyr supplementation similar to the increased urine melatonin metabolite, 6-sulfatoxymelatonin, to Trp supplementation: low concentration at baseline and increase with Trp supplementation. Although dopamine originates from the systemic neuronal cells and renal tubular cells as well as the brain, urine dopamine may still be a useful biomarker for the CNS dopamine metabolism for three reasons: (1) Tyr is transported into the peripheral neuronal cells as well as the brain by LAT1, (2) there are no isozymes known in Tyr hydroxylase (TH, EC 1.14.16.2), and (3) the similar profiles observed between urine dopamine and urine 6-sulfatoxymelatonin [12,13].

Since tetrahydrobiopterin (BH4) is a cofactor for Phe hydroxylase (PAH) as well as for TPH and TH which are the rate determining enzymes to synthesize serotonin and dopamine, respectively, we conducted the present study in adult individuals with PKU with three objectives: (1) to evaluate the direct effects of sapropterin dihydrochloride (the synthetic form of BH4) on brain TPH and TH by measuring the peripheral neurotransmitter biomarkers, melatonin and dopamine, (2) to determine if any synergistic effects are observed in melatonin and dopamine concentrations with concurrent use of LNAA and sapropterin (BH4), (3) to evaluate the relationship between blood Phe concentration and the peripheral neurotransmitter biomarkers.

## Methods

The study protocol was approved by the University of Southern California (USC) Health Science Institutional Review Board. Study subjects were recruited from our outpatient population based on the past history of compliance with medical instruction. Ten adult subjects with PKU were enrolled after obtaining written informed consent (Table 1). Table 1 presents subjects’ characteristics including range of Phe levels during 6 months preceding enrollment. All of the subjects were diagnosed by newborn screening. However, none of them restricted their diet to maintain blood Phe concentrations below 360 uM after relaxing their diet. All subjects prior to the study were on dietary therapy with either medical food products or BH4 therapy or both. The study was conducted in four 4-week phases. After an initial phase with LNAA supplementation (Phase 1, LNAA), LNAA was discontinued for the next 4-weeks (Phase 2, Washout). During the next phase they were treated with sapropterin at 20 mg/kg/day (Phase 3, BH4), after which supplementation of LNAA was added for the next 4 weeks (Phase 4, BH4+LNAA). All subjects consumed their regular diet with no medical food products or low protein products and avoided high protein foods. No dietary changes were made throughout the study. The number of LNAA tablets (PheBloc; Applied Nutrition, Cedar Knolls, New Jersey) taken daily, following the manufacturer’s recommendation, was based on the subject’s weight: body weight (kg) X 0.5 (maximum daily intake was 45 tablets), and provided the LNAA (mg/kg/d): Trp 30.6, Tyr 98.4, histidine 15.6, isoleucine 15.7, leucine 15.4, methionine 24.8, threonine 16.4, valine 16, and Phe 0. During Phases 3 and 4 subjects received 20 mg/kg/day of BH4, a dose reported to be transported into the brain [15, 16]. The study subjects stayed overnight at USC University Hospital at the end of each phase. All subjects were given the same protein-controlled meal during the overnight evaluation. Serum melatonin was measured every 2 hours from 7 p.m. to 7 a.m., and first void urine specimens were collected at 7 a.m. to measure dopamine and 6-sulfatoxymelatonin, to which 80–90% of melatonin is metabolized and is excreted into urine [17]. Plasma amino acids were obtained before dinner at 7 p.m. Serum and urine specimens were processed and kept in a freezer (-20C) until analyzed. Serum melatonin and urine 6-sulfatoxymelatonin were measured as described elsewhere [18]. Plasma amino acids and urine dopamine were analyzed by commercial laboratories. Blood specimens for serum melatonin were obtained and processed under dim light after 11 p.m. to avoid subjects’ eyes from being exposed to bright light, which potentially inhibits melatonin synthesis.

**Table 1:** Characteristics of Study Subjects.

Subjects	Age (y)	Gender	Clinical Phenotype	Genotype	Diet relaxed (age)	Phe levels (uM)	Highest Education
S1	32	male	moderate/classic	I65T/IVS4ntg>a	9	600–1200	College
S2	22	male	classic	S349P/IVS1nt5g>t	16	900–1500	College
S3	23	female	classic	R408W/IVS1nt5g>t	13	900–1800	High School
S4	26	male	classic	I65T/I65T	17	600–900	College
S5	45	male	classic	V387H/delT323	6	1200–1500	College
S6	22	male	classic	unknown	16	900–1200	High School
S7	24	male	classic	unknown	13	1200–1500	High School
S8	53	female	classic	Y277D/IVS12nt1g>a	6	1500–1800	High School
S9	40	female	classic	R408W/R408W	29	1200–1500	College
S10	52	male	classic	IVS12nt1g>a/IVS12nt1g>a	20	900–1200	High School

### Statistical Analyses

Serial concentrations of serum melatonin taken during the overnight stay were summarized as area under the curve of melatonin (AUC) vs. time, calculated by the trapezoidal formula. Additional outcome measures included concentrations of plasma Phe, and urinary concentrations of dopamine and 6-sulfatoxymelatonin. Prior to analysis study variables were log- transformed, as needed to normalize distributions. Comparisons among the 4 phases were made with repeated measures mixed models, with *post hoc* contrasts between the BH4 and Washout phases (Phase 3 vs. Phase 2) and between the BH4+LNAA and LNAA phases (Phase 4 vs. Phase 1), and also between Phase 1 and Phase 2 and between Phase 3 and Phase 4. Within-subject associations between plasma Phe and serum melatonin AUC as well as urine 6-sulfatoxymelatonin and dopamine were examined with equality of slopes mixed models. Statistical tests were 2-sided at a significance level of p<0.05, performed using SAS/STAT^®^ version 9.2 software. P-values between 0.05 and 0.10 were considered suggestive of a trend.

## Results

One subject (S10) was removed from the analysis since he received a medication that affects the CNS monoamine metabolism. The other nine completed all 4 phases, except for one subject (S3) who failed to collect a urine specimen in Phase 4. Table 2 summarizes serum melatonin (AUC), urine 6-sulfatoxymelatonin, plasma Phe, and urine dopamine levels for each study phase. There were no statistically significant differences in serum melatonin AUC, urine 6-sulfatoxymelatonin, plasma Phe or urine dopamine concentrations between Washout phase (Phase 2) and BH4 phase (Phase 3), nor between LNAA phase (Phase 1) and BH4+LNAA phase (Phase 4) in the study subjects as a whole. In contrast to BH4 therapy, serum and urine melatonin increased with LNAA in the study subjects as a whole: Phase 1vs. Phase 2 (p = 0.05 and 0.0002, respectively) and Phase 3 vs. Phase 4 (p = 0.01 and 0.0005, respectively). Urine dopamine also increased with LNAA: Phase 1 vs. Phase 2 (p = 0.002) and Phase 3 vs. Phase 4 (p = 0.0047).

**Table 2:** Serum melatonin AUC, urine 6-sulfatoxymelatonin, Phe, and urine dopamine levels at the end of each phase.

		Phase 1	Phase 2	Phase 3	Phase 4	Phase 2 vs 3 (p)	Phase 1 vs 4 (p)
**All subjects (N = 9)**	Serum Melatonin AUC	266.9±165.7	205.7±133.1	220.4±138.5	301.2±179.4	0.62	0.26
	Urine 6-sulfatoxymelatonin (ng/mg Cr)	14.5±9.0	8.2±4.8	8.6±5.7	13.2±9.3 (n = 8)	0.95	0.74
	Phenylalanine (uM)	1314.9±290.9	1436.6±223.8	1400±431.9	1190.9±367.8	0.62	0.11
	Dopamine (ug/gCr)	63.7±26.7	42.6±19.3	46.0±16.8	67.6±28.4 (n = 8)	0.57	0.78
**1) BH4 responder**	Serum Melatonin AUC	310.6±128.5	233.7±71.3	341.0±126.2	445.0±160.8	**0.07**	**0.03**
N = 3 (S1, S3, S4)	Urine 6-sulfatoxymelatonin (ng/mg Cr)	20.0±10.2	12.3±5.5	14.1±5.9	20.1±14.8 (n = 2)	0.16	0.49
	Phenylalanine (uM)	997.7±83.3	1250.7±185.8	895±76.9	741.0±254.4	**0.01**	**0.04**
	Dopamine (ug/gCr)	63.3±9.1	31±19.2	35.7±17.4	71.5±12.0 (n = 2)	0.69	0.69
**2) BH4 non-responder**	Serum Melatonin AUC	245.0±188.7	191.7±160.0	160.1±106.0	229.2±150.0	0.25	0.56
N = 6 (S2, S5, S6, S7, S8, S9)	Urine 6-sulfatoxymelatonin (ng/mg Cr)	11.8±7.9	6.1±3.1	5.8±3.2	10.9±7.2	0.55	0.57
	Phenylalanine (uM)	1473.5±205.0	1529.5±187.8	1652.5±258.0	1415.8±91.8	0.10	0.43
	Dopamine (ug/gCr)	63.8±33.2	48.3±18.1	51.2±15.4	66.3±33.1	0.70	0.73

Phase 1: LNAA supplement, Phase 2: Washout, Phase 3: BH4 therapy, Phase 4: BH4 and LNAA supplement.

Cr, creatinine; Data: mean ± SD.

There were no statistically significant differences in serum melatonin AUC, urine 6-sulfatoxymelatonin, plasma Phe or urine dopamine concentrations between Washout phase (Phase 2) and BH4 phase (Phase 3), nor between LNAA phase (Phase 1) vs. BH4+LNAA phase (Phase 4) in the study subjects as a whole. The study subjects (N = 9) are sub-grouped into two groups: BH4-responder (N = 3) and BH4 non-responder (N = 6).

Three subjects (S1, S3, and S4: BH4 responder) showed serum melatonin increase ≥30% in Phase 3 over Phase 2. In these 3 BH4 responders, plasma Phe concentrations were lower and melatonin AUC was higher in Phase 3 compared to Phase 2 (p = 0.01 and 0.07, respectively) and in Phase 4 vs. Phase 1 (p = 0.04 and 0.03, respectively), but no statistically significant differences were observed in urine 6-sulfatoxymelatonin or urine dopamine with supplementation of BH4. The remaining six subjects (BH4 non-responder) did not show any significant differences in these 4 variables with BH4 supplementation.

**Fig 1:**
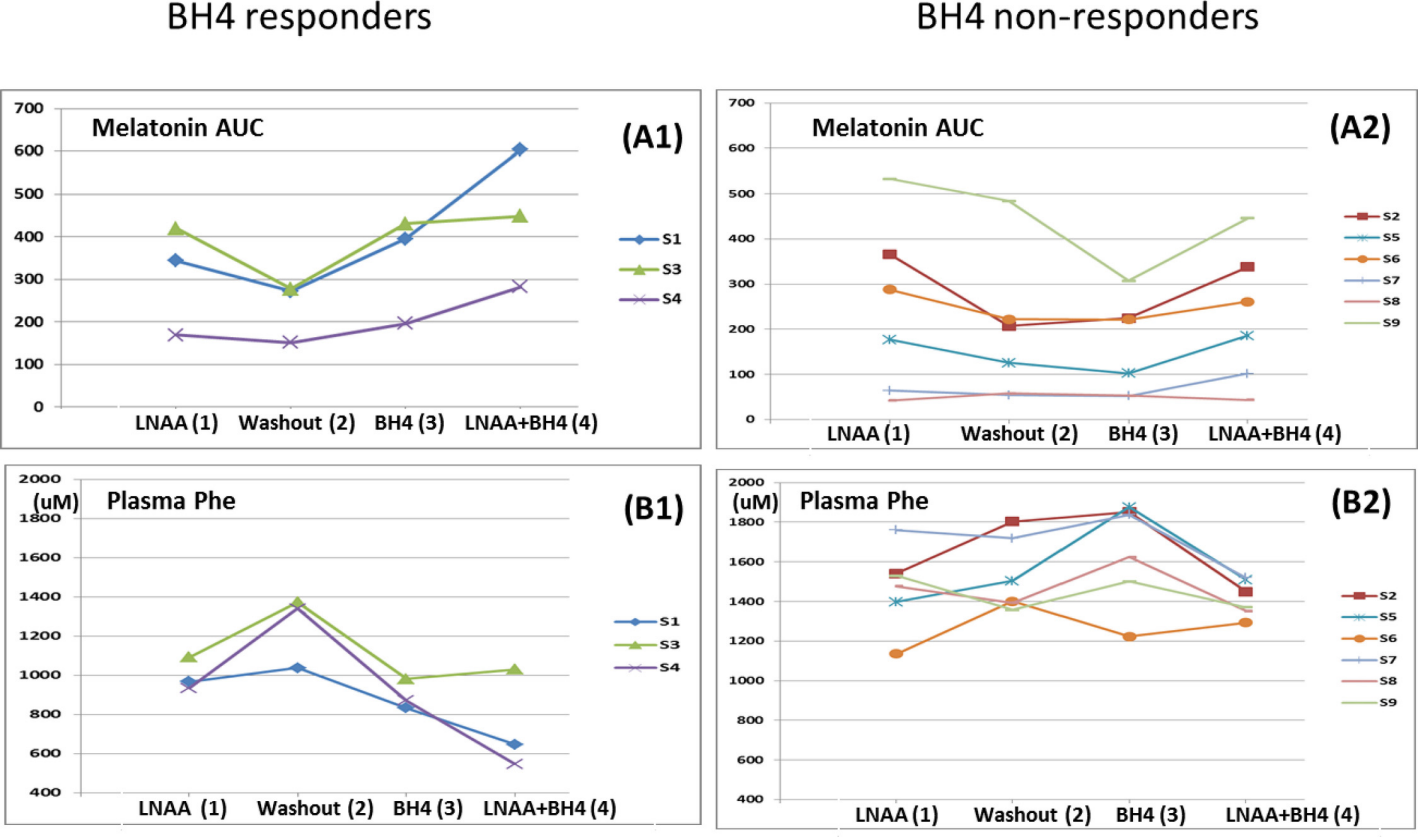
**Overnight serum melatonin AUC (A) and Plasma phenylalanine concentrations (B) over the 4 phases.** Phase 1 (LNAA), Phase 2 (Washout), Phase 3 (BH4) and Phase 4 (BH4 + LNAA). A1 and B1: BH4 responders. A2 and B2: BH4 non-responders. All BH4 responders showing significant decrease in plasma Phe levels, increased serum melatonin (Phase 3 vs. Phase 2 and Phase 4 vs. Phase 1). BH4 non-responders did not show increases in serum melatonin levels (Phase 3 vs Phase 2 and Phase 4 vs. Phase 1). All BH4 responders and S2, S5, S6, and S9 (4 out of 6 subjects) increased serum melatonin with LNAA (Phase 2 vs. Phase 1). All BH4 responders and S2, S5, S6, S7, and S9 (5 out of 6 subjects) increased serum melatonin with BH4 and LNAA over BH4 only (Phase 4 vs. Phase 3).

**Fig 2:**
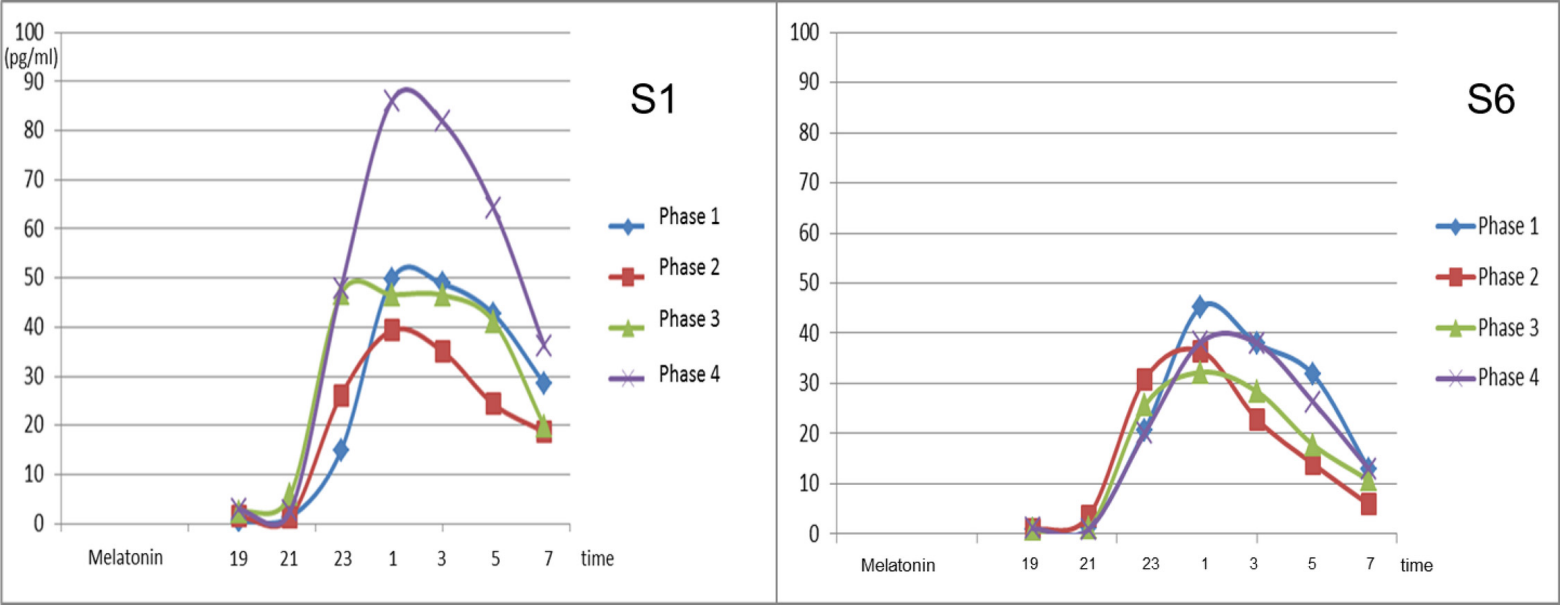
Overnight (7 pm to 7 am) serum melatonin levels. Phase 1: LNAA supplement, Phase 2: Washout, Phase 3: BH4 therapy, Phase 4: BH4 and LNAA. Left: melatonin responder to BH4 (S1), Right: melatonin non-responder to BH4 (S6). Serum melatonin (pg/ml).

1) Serum Melatonin (Fig 1A1 and 1A2, Fig 2)

Serum melatonin levels (AUC) at all phases from all subjects are shown in Fig 1A1 and 1A2. The three BH4 responders (S1, S3, and S4) increased in serum melatonin by 45%, 55% and 30%, respectively, in response to sapropterin therapy (A1). The remaining six subjects showed a minimal increase (S2), no changes (S6, S7, and S8), or decrease (S5, S9) in serum melatonin (A2). In one BH4 responder (S1) serum melatonin increased even more when BH4 was added to LNAA (75% vs 45%), indicating synergistic effects between BH4 and LNAA supplementation. Overnight serum melatonin in S6 are also shown as a melatonin non-responder to BH4 for comparison to S1 as a responder (Fig 2). Three (S1, S4, and S7) out of nine subjects showed the synergistic effects of sapropterin and LNAA on serum melatonin AUC by 76%, 67%, and 59% increase, respectively (Phase 4 vs. Phase 1). One subject (S9) showed a 36% decrease after sapropterin therapy.

2) Plasma Phe levels (Fig 1B1 and 1B2)

Plasma Phe concentrations from all phases in each study subject are shown in Fig 1B1 and 1B2. The subjects (S1, S3, and S4) who responded to sapropterin with an increase in serum melatonin also showed decreases in plasma Phe concentrations (20%, 29%, and 35%, respectively; Phase 3 vs. 2). The subjects (S1, S4, and S7) with synergistic effects on serum melatonin showed decreased plasma Phe concentrations as well (33%, 41%, and 14%, respectively; Phase 4 vs. Phase 1). S6 showed 13% reduction in plasma Phe concentrations in Phase 3 without synergistic effects in Phase 4.

**Fig 3:**
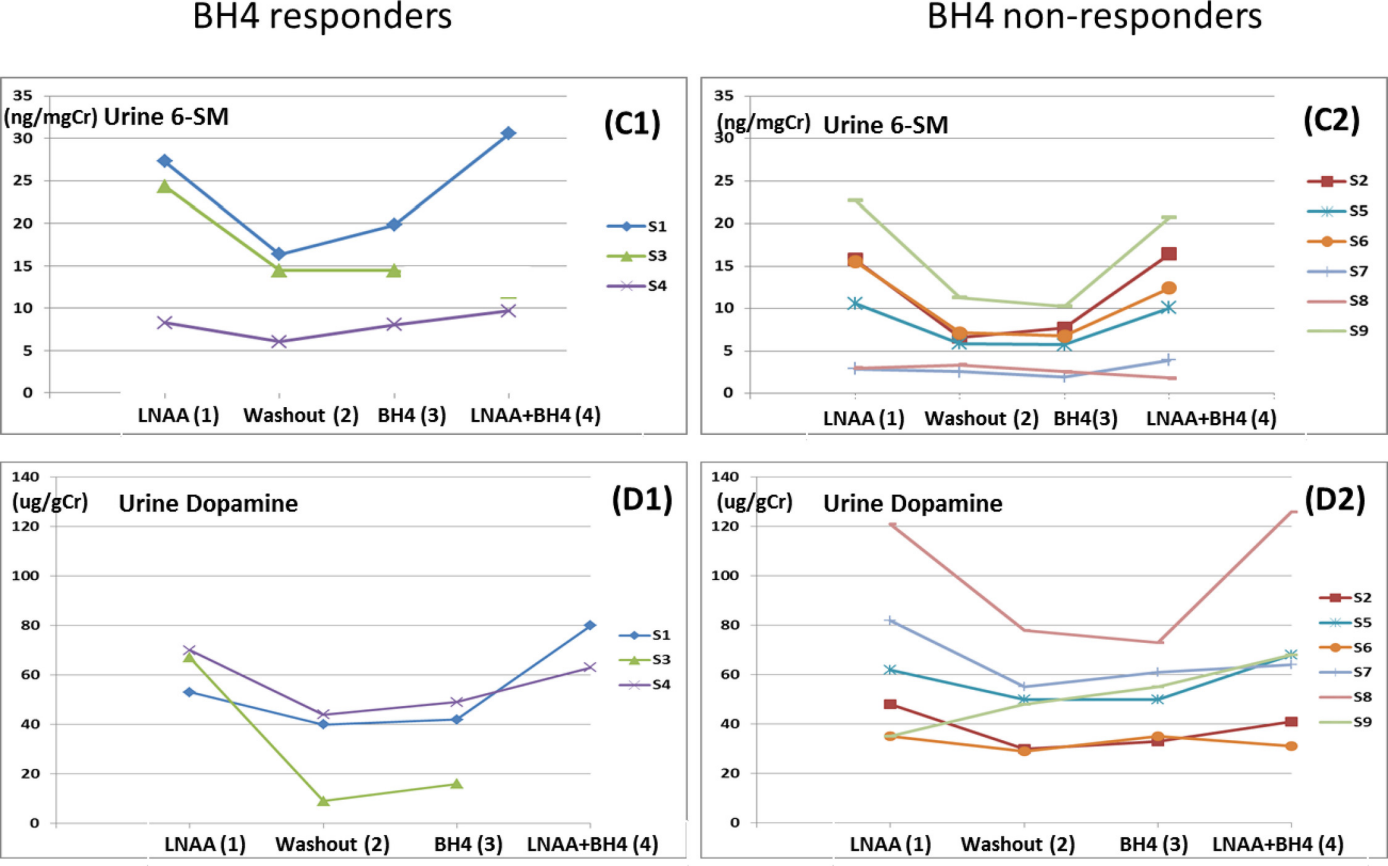
Urine 6-sulfatoxymelatonin (C) and Urine dopamine concentrations (D) over the 4 phases. Phase 1 (LNAA), Phase 2 (Washout), Phase 3 (BH4) and Phase 4 (BH4+LNAA). C1 and D1: BH4 responders. C2 and D2: BH4 non-responders. The data point at Phase 4 in S3 is missing due to sample collection failure.

3) Urine Melatonin (Fig 3C1 and 3C2)

Two out of 3 BH4 responders showed increase in urine melatonin in Phase 3 over Phase 2. The data point at Phase 4 in S3 is missing due to sample collection failure. Six BH4 non-responders showed no increase in urine melatonin with BH4 supplementation.

4) Urine Dopamine (Fig 3D1 and 3D2)

Profiles of urine dopamine concentrations were similar to those of urine 6-sulfatoxymelatonin (C1 and C2) except for S9 whose dopamine concentration was higher in Phase 2 than in Phase 1.

**Fig 4:**
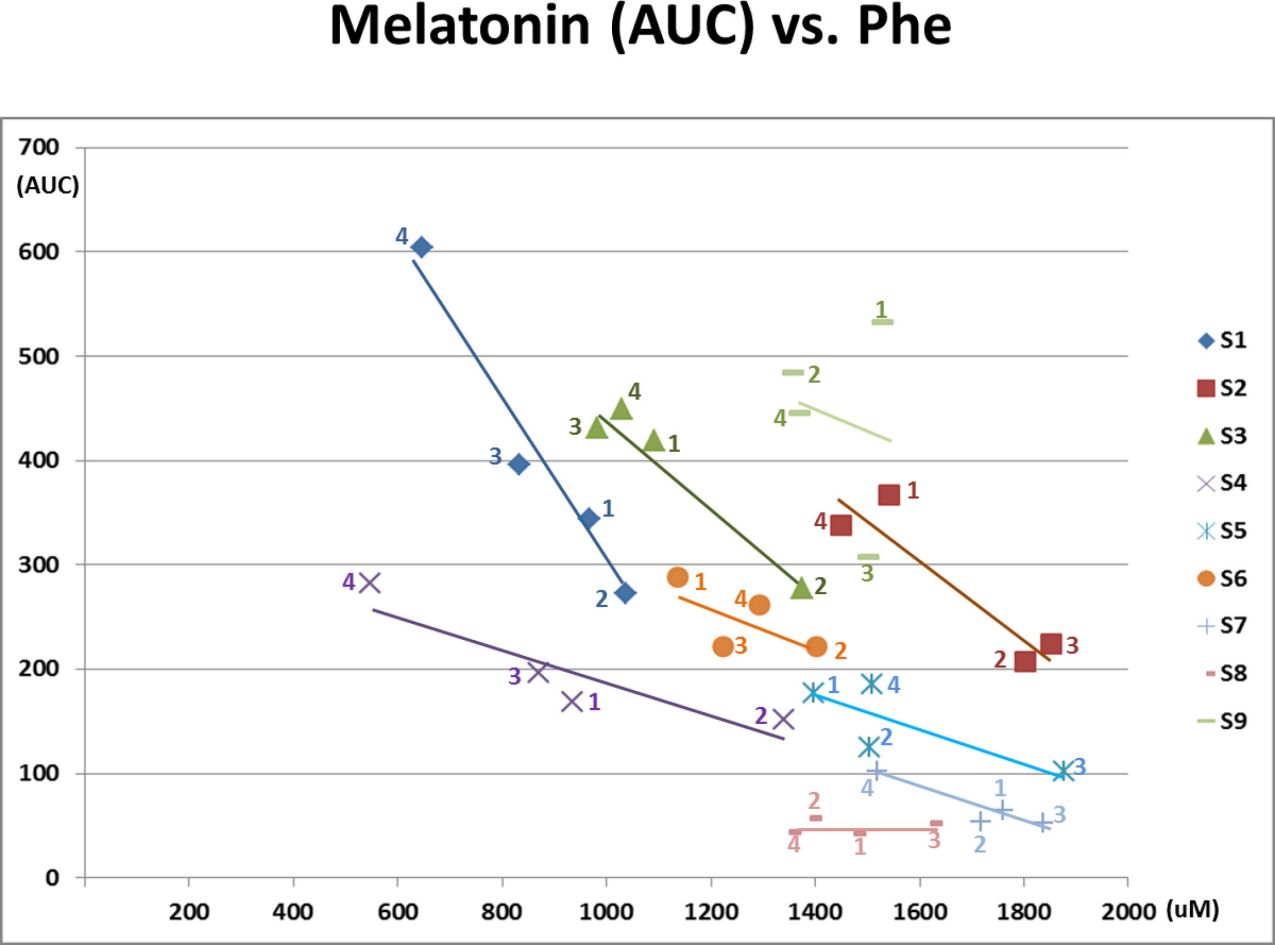
Serum Melatonin (AUC) vs. Plasma Phenylalanine. The number of each data point indicates the study phase. Negative correlations with plasma phenylalanine concentrations and serum melatonin AUC (p = 0.0005) is shown. Except for S8, a linear negative regression line is drawn for each subject. The slope of linear regression line differs among the 9 subjects (p = 0.066).

**Fig 5:**
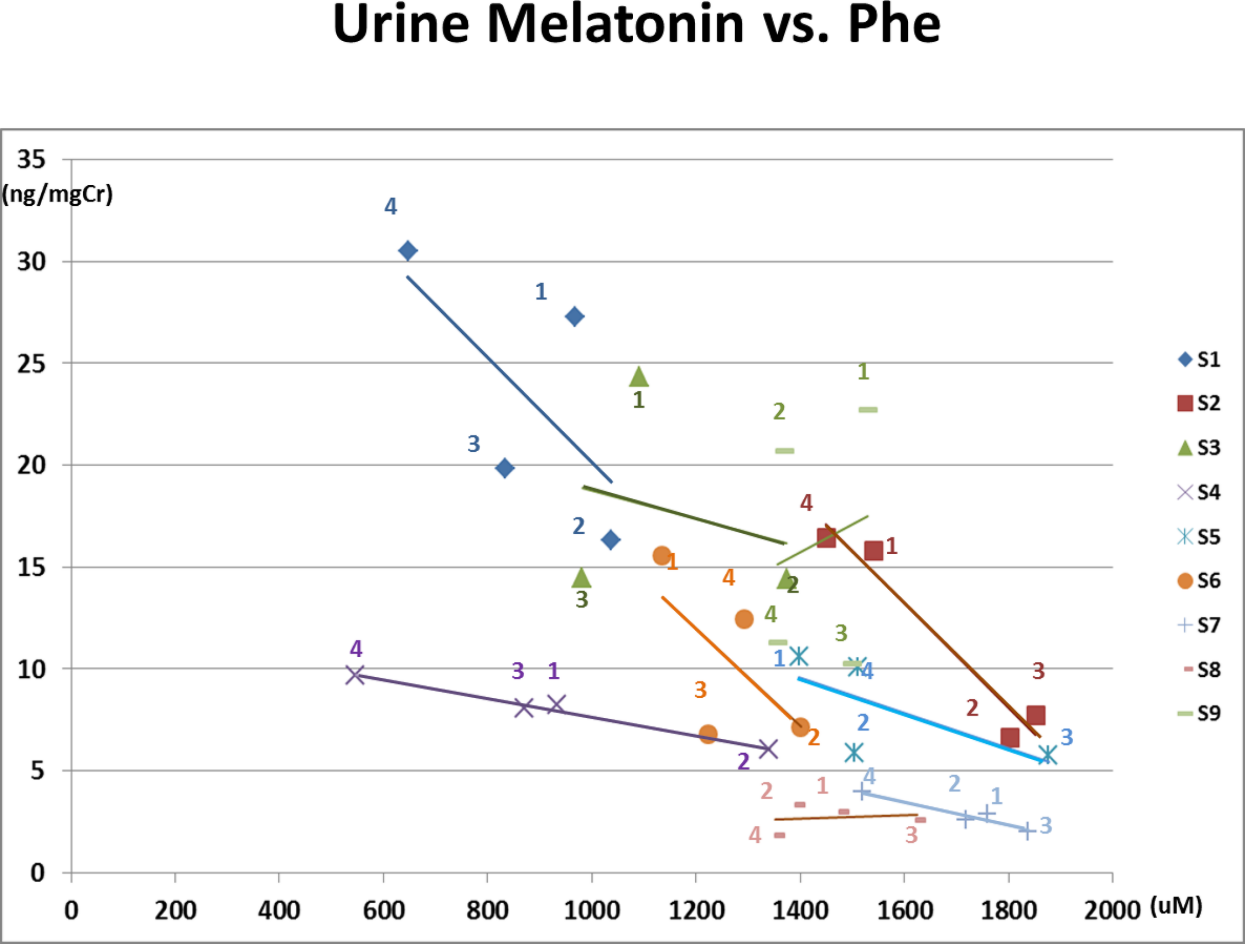
Urine 6-Sulfatoxymelatonin vs. Plasma Phenylalanine. The number of each data point indicates the study phase. Negative correlations with plasma phenylalanine concentrations and urine 6-sulfatoxymelatonin (p = 0.04) is shown. Except for S8 and S9, a linear negative regression line is drawn for each subject.

5) Plasma Phe vs. Serum Melatonin AUC and Plasma Phe vs. Urine 6-sulfatoxymelatonin (Figs 4 and 5)

Fig 4 shows that serum melatonin AUC values varied widely at a particular plasma Phe concentration among the study subjects, and that the association between plasma Phe concentrations and serum melatonin AUC also varied by subject. The mixed model analysis of the relationship between AUC and Phe showed a significant negative slope (-0.38, p = 0.0005), with a trend toward differing slopes among subjects (p = 0.066). The slopes were negative for 8 of the 9 subjects, ranging from -0.15 (S5) to -0.82 (S1), while one (S8) showed no change in melatonin AUC with change in Phe, slope = 0.01. Since AUC utilizes all seven overnight assessments, it likely more accurately reflects melatonin synthesis than a single urine 6-sulfatoxymelatonin measurement. Even so, a significant negative association overall between plasma Phe and urine 6-sulfatoxymelatonin was also identified (Fig 5, p = 0.040).

**Fig 6:**
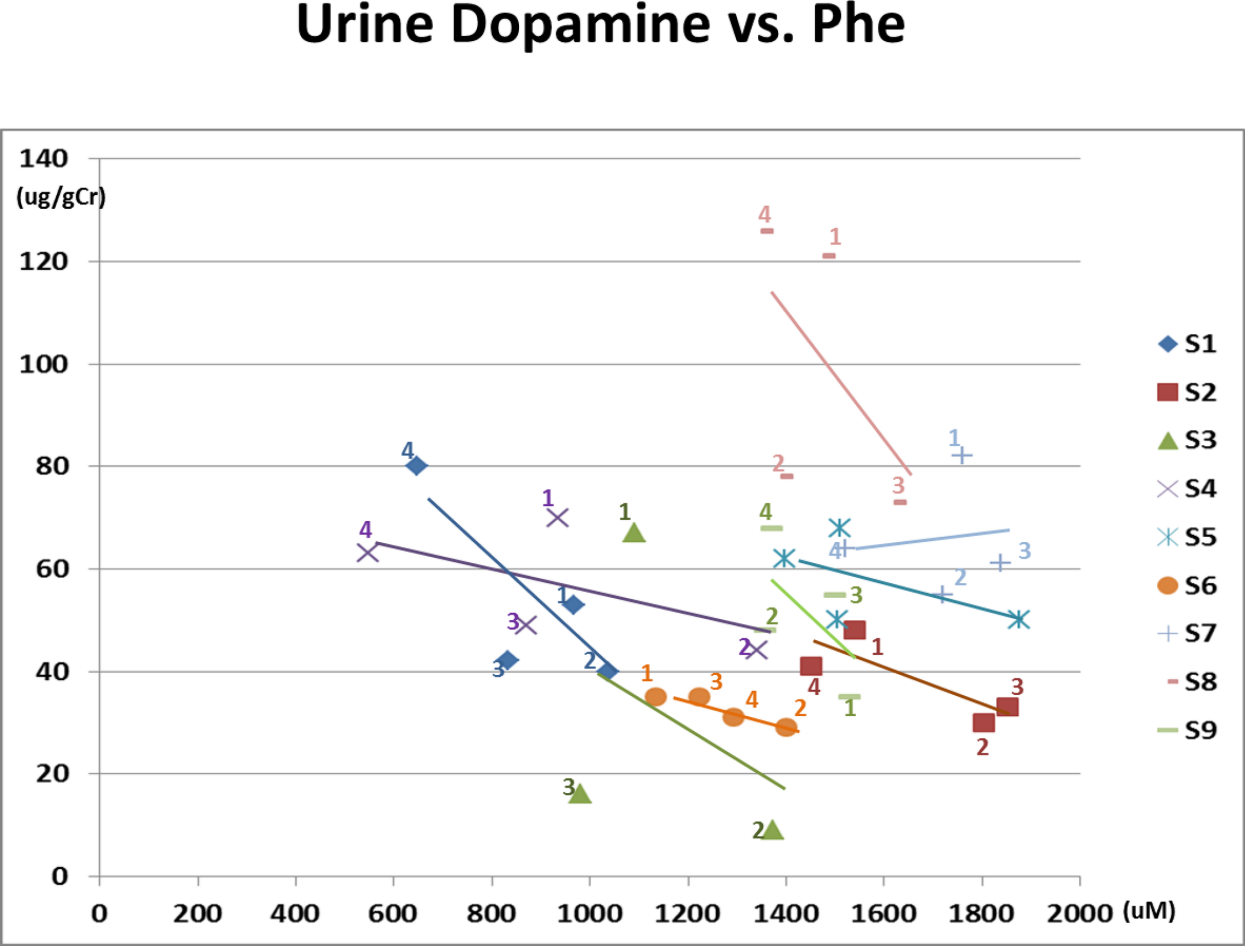
Urine dopamine vs. Plasma phenylalanine. The number of each data point indicates the study phase. Negative correlations with plasma phenylalanine concentrations and urine dopamine (p = 0.047) are shown. Except for S7, a linear negative regression line is drawn for each subject.

6) Plasma Phe vs. Urine Dopamine (Fig 6)

Urine dopamine concentrations are again quite variable at a particular plasma Phe concentration among the subjects. There was a negative association overall between plasma Phe and urine dopamine (p = 0.047).

## Discussion

PKU is caused by a deficiency of PAH which is expressed in the liver but not in the CNS. The neurocognitive and neuropsychological symptoms associated with PKU are likely due to the unique environment in the CNS with high blood Phe and relatively low concentrations of the other amino acids. This may contribute to fetal microcephaly in maternal PKU syndrome through decreased protein synthesis in the rapidly growing stage and to neurocognitive and neuropsychological symptoms after CNS development and growth are completed, possibly due to abnormal neurotransmitter metabolism [3, 19]. Butler et al. reported direct evidence of low CSF concentrations of HVA, a dopamine metabolite, and 5HIAA, a serotonin metabolite, in individuals with PKU prior to dietary treatment and subsequent improvement in these metabolites after dietary treatment [5]. Lykkelund reported negative associations between CSF HVA and 5HIAA and blood Phe in PKU by comparing concentrations before and after dietary therapy [14]. Improvement of reaction time was also observed accompanied by significant increases in these neurotransmitters [14].

Burton et al. recently reported improvement in symptoms of attention deficit-hyperactivity disorder and impairments in executive functioning in individuals with PKU who responded to sapropterin therapy with reduction of blood Phe concentrations, suggesting involvement of the monoamine neurotransmitters, serotonin and dopamine, in neurocognitive and neuropsychological symptoms in PKU [20]. Our study showed consistent findings with improvement of plasma melatonin concentrations after sapropterin therapy with reduction of blood Phe, but no improvement without the reduction of blood Phe concentrations. These findings suggest that brain serotonin metabolism improved not through the direct effects of sapropterin on TPH but secondarily to reduced blood Phe concentrations, likely resulting in increased Trp transport into the brain. When LNAA was supplemented, improvement of serum and urine melatonin as well as urine dopamine levels was observed although no significant changes were observed in plasma Phe concentrations (Table 2, Phase 1 vs. Phase 2). This observation is consistent with our previous studies, indicating increased transport of Trp and Tyr into the brain at LAT1 by increasing blood Trp and Tyr concentrations [12,13]. Synergistic effects on improvement of brain monoamine neurotransmitters by LNAA and sapropterin, which were observed in BH4 responders, are likely due to increased blood concentrations of Trp and Tyr by LNAA supplementation and reduction of blood Phe concentration by sapropterin.

A wide range of serum melatonin AUC as well as urine 6-sulfatoxymelatonin concentrations, which likely reflect brain serotonin synthesis, was observed at a particular blood Phe concentration within this small number of study subjects. One individual (S9) had the highest melatonin AUC (within the range reported for non-PKU adults [12]) at Phe > 1200 uM in the Washout phase. Serum melatonin and blood Phe had a clear negative association in the majority of study subjects, suggesting serotonin synthesis in the CNS is more suppressed by higher blood Phe concentrations, with differing slopes among the subjects indicating significant inter-individual variation in serotonin metabolism in response to blood Phe. Based on these findings, blood Phe concentration alone cannot be used to evaluate brain serotonin metabolism in individuals with PKU. The wide variation of serotonin metabolites among study subjects may suggest involvement of LAT1 and TPH1 polymorphism which most likely affect LNAA transport into the CNS through LAT1 as well as inhibition of TPH activities by Phe. Although establishing the reference range of serum melatonin AUC requiring an overnight stay at the hospital is impractical, it is nevertheless feasible and critically important to establish reference ranges of 6-sulfatoxymelatonin in first void urine by age and gender. Serum melatonin (AUC) and urine melatonin levels showed no discrepancy throughout the study except for two data points: urine melatonin levels in S3 in Phase 3 and in S9 in Phase 2 showing lower concentrations than expected when compared to the serum levels. Although serum melatonin AUC reflecting 7 data points is likely more accurate than once urine melatonin concentration point, the cause of this discrepancy was unclear.

Urine dopamine concentrations at a given blood Phe concentration also varied widely among study subjects along with a negative association between blood Phe and urine dopamine (Fig 6). Establishing reference ranges for dopamine concentrations in first void urine could also be useful as an indirect marker to reflect CNS dopamine metabolism.

Manti el al. recently reported the occurrence of psychiatric disorders in which anxiety and withdrawal are the most frequent self-reported symptoms, is not associated with either the lifelong or concurrent quality of metabolic control, and that even individuals with good metabolic control (Phe<500uM) in the first 11 years of life showed a higher frequency of psychiatric diagnoses compared to healthy controls [21]. This indicates the involvement of factors other than blood Phe concentrations affecting neurocognitive and neuropsychiatric symptoms.

Nardecchia et al. reported that developmental trajectories in some cases of early treated PKU were independent from metabolic control, suggesting individuals vary in their vulnerability to Phe [22]. Our study suggests that neurotransmitter metabolism may account for this individual vulnerability to Phe.

## Conclusion

Although concentrations of neurotransmitters, i.e., serotonin, dopamine or their derivatives 5HIAA and HVA in CSF were not directly measured and compared to concentrations of the peripheral biomarkers, this study supports the hypothesis that peripheral concentrations of melatonin and dopamine, which may reflect serotonin and dopamine metabolism in the CNS, respectively, might be used to evaluate deficiencies of these monoamine neurotransmitters in the CNS. The wide variation of the peripheral biomarker levels at a particular blood Phe concentration, and also the differing responses among individuals in the peripheral biomarkers to Phe concentrations indicates that blood Phe concentrations alone cannot be used to evaluate brain serotonin and dopamine metabolism. Evaluation of urine melatonin and dopamine along with blood Phe concentrations may identify neurotransmitter deficiencies and allow clinicians to determine the most effective individualized management, either by providing more Trp and Tyr through medical food products and/or LNAA, or by reducing natural protein intake to lower blood Phe levels and thus improve serotonin and dopamine metabolism. This individualized, targeted management of PKU may reduce long-term neurocognitive and neuropsychological problems. Establishing reference ranges for these peripheral biomarkers is essential. Larger, long-term follow up studies are needed to evaluate this hypothesis.

## Supporting Information

S1 File Study Data. All of the data obtained from this study including plasma tyrosine, phenylalanine, tryptophan, urine 6-sulfatoxymelatonin, urine dopamine, and serum melatonin AUC are shown.
(XLSX)
